# Medical Department of Transylvania University*Numerous handbills, pamphlets and newspaper communications.

**Published:** 1837

**Authors:** 


### Art. VI.—Medical Department of Transylvania University.*

It is not our design to plunge into the controversy which has unhappily arisen, among our *quondam* colleagues, in "Old Transylvania;" but her numerous and respectable graduates, many of whom are readers of this Journal, and live in remote quarters of the interminable West, have a right to expect some information (*ætiological* if no more) on the convulsions which have agitated her. We have been constant, and (hope) impartial readers of the various publications, and have, moreover, had opportunities of conversation with some of our friends of both parties; we have, thus, as we suppose, acquired such an insight into the matter, as qualifies us to write upon it a historical paragraph.

It appears that, consulting what they regarded as the interest of the school and the profession, rather than that of the people of Lexington, by whose liberality it had been founded and cherished, the professors, early in the year 1836, came to the unanimous and confidential conclusion, that it ought to be transferred to Louisville; there to be and remain a branch of Transylvania University, bearing her name and acknowledging her sceptre. Thus a *new* school was not contemplated, but a better location; and no other institution could, as they supposed, be reared up at Lexington, as there was no legal

---

* Numerous handbills, pamphlets and newspaper communications.

authority for such an establishment, but that confided to the Transylvania trustees. It is admitted, that all the professors had the impression, that the trustees, or, at all events, the legislature, had the power to direct this transfer; and it seems to have been hoped, that the people of Lexington, of whom the board is chiefly composed, would acquiesce. In the course of the following autumn, the subject was broached to certain influential citizens of Louisville, and assurances were given by them, that the city authorities would make a liberal appropriation for the accommodation of the Faculty. By this time, however, the project had become known in Lexington, where it created a degree of excitement as violent as it was natural. This excitement seems not to have been fully anticipated by the professors, and was, evidently, most unacceptable to such of them, as had spent their lives among those in whom it had been raised. The trustees, several of whom are eminent jurists, immediately arrayed themselves against the measure; and without hesitation declared, that the *legislature had not the power to abolish any of the franchises of the charter; that if the object were prosecuted, the whole Faculty should be dismissed, aud another appointed; and, that Lexington would maintain a school, notwithstanding Louisville might establish one.* Under this state of things, the professors separated from each other, in their future plans. Three of them resolved to remain in Lexington, and three to prosecute the formation of a school in Louisville, under a new charter. A very violent state of personal animosity now sprung up, appeals were made to the board of trustees, charges and counter charges were preferred, and recriminations were piled on criminations, proportionately to the friendships previously existing.

In the month of March last, the board took the matter seriously in hand, and after such an inquiry as they conceived proper, dismissed one of the professors, and declared the chairs of all the rest vacant. Soon afterwards, the three, Drs. Dudley, Richardson and Short, who had seceded from the Louisville project, were reinstated, and the other three, Drs. Caldwell, Cooke, and Yandell were left to its prosecution.

Under our subsequent head of original intelligence, the reader will find a notice of what has since been done, by the respective parties, in the work of re-organization. D.

